# High Overlap in Niches and Suitable Habitat Between an Imperiled and Introduced Cottontail

**DOI:** 10.1002/ece3.71083

**Published:** 2025-03-17

**Authors:** Kathryn E. Bischoff, Danielle Katz, Chadwick D. Rittenhouse, Tracy A. G. Rittenhouse

**Affiliations:** ^1^ Department of Natural Resources and the Environment University of Connecticut Storrs Connecticut USA

**Keywords:** habitat suitability, imperiled species, introduced species, maxent, niche overlap, rare species conservation

## Abstract

When introduced species invade new environments, they often overlap with native species currently occupying those spaces, either spatially through suitable habitat or environmentally through their realized niches. The goal of this research is to determine the overlap between native New England cottontail ( *Sylvilagus transitionalis* ) and introduced eastern cottontail ( *Sylvilagus floridanus* ) to identify potential areas of invasion by the eastern cottontail and potential areas of refuge for the New England cottontail from the eastern cottontail (Connecticut, USA). Using presence data from a regional, standardized monitoring protocol, we developed habitat suitability models using Maxent and conducted niche overlap analyses using environmental principal component analysis. We used several covariates that reflected proximity to habitat characteristics, such as young forest, shrubland, and understory, as well as proximity to threats, such as development. We also included topographic and climatic covariates. We used the Guidos software to categorize the spatial arrangement of young forest, shrubland, and understory vegetation. We found that the overlap in both niches and suitable habitat was high for two species. Only areas of low precipitation and high elevation shifted niches in favor of the New England cottontail. We also found that habitat suitability for the New England cottontail was higher when patches of mature forest without understory were within complexes of young forest, shrubland, and mature forest with understory. Increasing habitat heterogeneity could improve the habitat suitability of existing patches or create new patches for New England cottontail. However, habitat management alone is likely not enough to discourage eastern cottontail; thus, direct species management, such as removal of eastern cottontail and augmentation of New England cottontail populations, should be explored.

## Graphical abstract

The rare and declining New England cottontail ( *Sylvilagus transitionalis* ) faces increased competition from an introduced competitor, the eastern cottontail ( *Sylvilagus floridanus* ). We developed species distribution models using Maxent and niche overlap models using principal component analysis to identify spatial and environmental niche overlap. We found high niche overlap between the two species and that habitat highly suitable for New England cottontail is also highly suitable for eastern cottontail, but increasing habitat heterogeneity may improve habitat suitability for New England cottontail.

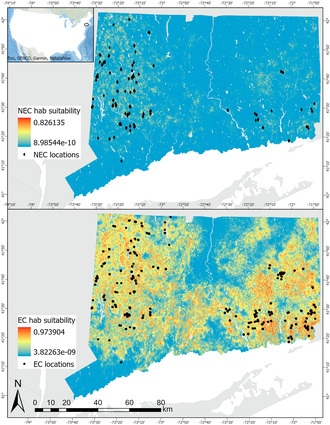

## 1 Introduction

Simultaneously understanding species habitat suitability and niches can be useful for the conservation of rare species in the face of global change (Quiroga and Souto 2022), especially when introduced competitors are present (Pascual‐Rico et al. 2020). Habitat suitability models often address different aspects of niche theory (Hirzel and Le Lay 2008). Grinnell (1917) established the connection between niches and habitat suitability, where he recognized a species niche is determined by factors regarding habitat quality, such as climate, food, and soil. This connection between niche theory and habitat suitability is the foundation for many species distribution modeling techniques (Richardson and Whittaker 2010), having applications in species conservation (Quiroga and Souto 2022), predicting niche changes in response to changing climate conditions (Petitpierre et al. 2012), and predicting the invasion of non‐native species (Santamarina et al. 2023; Ramirez et al. 2024). Species can share both geographic and environmental space, leading to overlap in niches and habitat suitability. Quantifying the overlap in both environmental and geographic space can aid native species conservation.

Despite consistent conservation efforts over the last few decades, the rare New England cottontail (*Sylvilagus transitionlis*) is still declining due to habitat loss and co‐occurrence with an introduced competitor, the eastern cottontail ( *Sylvilagus floridanus* ; Litvaitis et al. 2008; Kovach et al. 2022). The New England cottontail was once found throughout eastern New York and New England wherever young forest, shrubland, and dense understory habitats were abundant (Nelson 1909). However, due to forest maturation and forest loss to urban development, young forest and shrubland habitat declined substantially during the 1900s (Brooks 2003; Litvaitis 2003; Lorimer and White 2003) and subsequently, extirpations have occurred at state (Vermont, Rhode Island) and population levels throughout the remaining range (Litvaitis et al. 2006; Fenderson et al. 2011, 2014; Brubaker et al. 2014; Rittenhouse and Kovach 2020).

Conservation efforts for New England cottontail have included population augmentation from wild and captive populations (Bauer et al. 2020; Ferry 2023), translocation of wild populations (Eline, Cohen, McGreevy Jr., et al. 2023), and using vegetation management to create and maintain suitable habitat (Kovach et al. 2022; Eline, Cohen, Whipps, et al. 2023). Population augmentation and translocation could increase the genetic diversity of the five genetically isolated regional populations (Fenderson et al. 2011) and have increased patch abundance and dispersal (Bauer et al. 2020). Maintaining young forest, shrubland, and mature forest with dense understory is crucial for New England cottontail because the high stem density provides cover and protection from predators (Barbour and Litvaitis 1993; Litvaitis 1993, 2001; Cheeseman et al. 2018, 2021; Cheeseman, Cohen, Whipps, et al. 2019) and shrubland with higher vegetation height increases New England cottontail occupancy probability while decreasing eastern cottontail occupancy probability (Bischoff et al. 2023a). Concurrent with landscape‐level changes in habitat, range expansion of eastern cottontail into the New England cottontail range has complicated New England cottontail management efforts by displacing New England cottontail from young forest and shrubland habitat (Cheeseman et al. 2021) through interspecific competition (Probert and Litvaitis 1996; Cheeseman et al. 2018; Bischoff et al. 2023b).

Eastern cottontail invading the range of the New England cottontail is an extraordinary case of invasion effects on rare species. Before 1899, the only cottontail found in New England was the New England cottontail (Johnston 1972). The first eastern cottontail was introduced to mainland New England in the early 1900s, and subsequently, private hunting clubs and state agencies continued to introduce eastern cottontails for several decades (Johnston 1972). In less than 100 years since the first confirmed eastern cottontail occurrence, the eastern cottontail has become ubiquitous across most of the New England cottontail range (McGreevy Jr. et al. 2021). As the introduced competitor's range expanded, the New England cottontail range and occupancy within its range declined (Litvaitis et al. 2006; Rittenhouse and Kovach 2020). The conservation concern surrounding New England cottontail allowed New England cottontail to be a highly researched species, and thus, habitat associations for both species in the northeastern United States are largely understood. The factors that encourage New England cottontail occupancy and survival while potentially discouraging eastern cottontail populations include vegetation height above 0.5 m (O'Connor 2015; Cheeseman et al. 2018, 2021; Bischoff et al. 2023a; Bischoff et al. 2023b) and moderate canopy closure (Buffum et al. 2015). However, these habitat conditions are ephemeral and dynamic across the landscape; thus, the location of where these habitat conditions currently occur or might be created through vegetation management has yet to be determined.

Given the rapid expansion of eastern cottontail and concurrent decline of New England cottontail, we used habitat suitability models and niche overlap analyses to investigate overlap between the native New England cottontail and introduced eastern cottontail spatially (i.e., suitable habitat) and environmentally (i.e., niches). Our goal was to identify spatial and environmental areas suitable for New England cottontail but not eastern cottontail. To do this, we assessed how similar or equivalent the two species' niches were and habitat uniquely suitable for each species. The hypothesis we tested for niche overlap was that New England and eastern cottontail niches are more equivalent and similar than random. Spatially predicting overlap allowed us to identify future areas of invasion for eastern cottontail as well as new areas for New England cottontail sampling and habitat conservation.

## 2 Methods

### 2.1 New England Cottontail and Eastern Cottontail Presence Data

We used presence data collected by the New England Cottontail Regional Monitoring Program from 2016 to 2022 in Connecticut. The Regional Monitoring Program was established in 2015 as a collaboration between all states with extant populations of New England cottontail (Connecticut, Maine, Massachusetts, New Hampshire, New York, and Rhode Island), where biologists from each state collected cottontail fecal pellets at designated sites. Observers collected pellets each winter (from November to April) during conditions that maximize pellet detection and quality: snow‐covered ground, low temperatures, and 2–4 days after a snowfall or high wind event (Kovach et al. 2003; Brubaker et al. 2014; Whipps et al. 2020). To collect pellets within sites, observers walked parallel transects that were spaced at least 30.0 m apart. Observers chose the direction of the transects within each site and consistently used the same direction within a site, but the transect direction could differ between sites. Observers searched for a pile of cottontail pellets up to 15.0 m on either side of the transect, and once a pellet pile was found, observers collected at least one pellet from the pile (hereafter referred to as sample) and placed it into a vial. Observers recorded GPS coordinates of each sample collected. Observers then walked at least 30 m before collecting the next sample to ensure that samples were collected from throughout the site (Rittenhouse and Kovach 2020). Following this field protocol, an average of 0–10 samples were collected at each site for each visit.

Fecal mitochondrial DNA analysis with barcoding of diagnostic characters was used to extract high‐quality DNA and identify species (Sullivan et al. 2019; Whipps et al. 2020). DNA extraction and species identification occurred in over 99% of the samples (Sullivan et al. 2019), but when DNA was not extracted from the sample, we excluded the sample from the analysis. We cross‐referenced species identification with the location of the pellet to create a presence dataset of both New England and eastern cottontail pellets within sites in Connecticut. Observers recorded when a species was absent from a site, not a specific location, so we could not include absence data within the models.

Sites were sampled within defined New England cottontail focus areas; thus, we accounted for spatial autocorrelation using the “spThin” package (Aiello‐Lammens et al. 2015) in R 4.3.1 (R Core Team 2023). We used a thinning distance of 10.0 m to align with the fine spatial resolution of the environmental predictors (10.0 m cell size). Samples within 10.0 m of each other were randomly selected for inclusion in the data set. The total occurrences before thinning for New England cottontail was 2086 and eastern cottontail were 4285. After thinning and removing multiple occurrences within one cell, the number of occurrences for New England cottontail was 1735 and eastern cottontail was 3629, a reduction of 16.83% and 15.31%, respectively. The average geographic distance between thinned locations was 49.57 m for New England cottontail locations and 33.81 m for eastern cottontail locations. We used the thinned datasets for all analyses. Since we used data collected in the winter only, models described New England cottontail and eastern cottontail winter distribution and niches. Winter is the time of year when cottontail survival is typically lowest, depending on body condition (Cheeseman et al. 2021), due to limited resources and harsher weather conditions.

### 2.2 Predictor Variables

We included environmental predictors that encompassed land cover, climate, and topography attributes measured across the entire state of Connecticut. We used building footprints as a proxy for development (Microsoft 2019). We used the 1.0 m resolution Coastal Change Analysis Program land cover map to extract mixed forests (National Oceanic and Atmospheric Administration 2020). We used a Young Forest and Shrubland Vegetation Map to identify different types of young forest and shrubland vegetation within Connecticut (Rittenhouse et al. 2022). The map used ecological processes (succession, disturbance, regeneration, and hydrology), vegetation height, percent vegetation cover by height category, previous land cover type, and time since disturbance to classify different types of young forest and shrubland vegetation (Rittenhouse et al. 2022). We combined the regenerating forest and regenerating clearcut classes into one predictor (hereafter called regenerating forest) for this analysis. We also used an understory vegetation map to identify locations of greenbrier ( *Smilax rotundifolia* ), Japanese barberry ( *Berberis thunbergii* ), mountain laurel ( *Kalmia latifolia* ), and other mixed invasive understory species within deciduous forests of Connecticut (Yang et al. 2023). Using the National Wetlands Inventory (US Fish and Wildlife Service 2020), we extracted wetlands and a specific list of forested/shrub wetland types found in sites known to be occupied by New England cottontail (Rittenhouse et al. 2022). We calculated elevation, northern and eastern aspects, and slope from 30.0 m elevation tiles (National Aeronautics and Space Administration 2000). We used 1.0 km resolution annual total precipitation (mm) data (Thornton et al. 2020) and averaged annual total precipitation across multiple years within the study period (2016–2019). Annual precipitation data was only available until 2019, so we could not measure total annual precipitation for more recent years of the study. We removed hydrological features from all predictors to avoid identification of lakes, ponds, rivers, and streams as suitable habitat (Elith et al. 2011). To improve the performance of subsequent analyses, we converted all land cover predictors to continuous predictors by creating distance surfaces with an offset of 1.0 to replace all zero distance values, since zero‐inflated data can bias principal component analysis (PCA) results (Hellton et al. 2021).

Most of the predictors have a resolution of 10.0 m or less; however, some of the climate and topographic variables are only available at coarser resolutions. To create robust and comprehensive species distribution models and include all types of covariates (ecological, climatic, and topographic) that likely influence the suitability of cottontail habitat, we resampled all covariates to 10.0 m. We chose a 10.0 m resolution because this was the finest resolution possible with the computational ability available, the spatial resolution used to create the young forest and shrubland and understory covariates (Rittenhouse et al. 2022; Yang et al. 2023), and the resolution to avoid degrading fine resolution predictors. We included covariates at a coarser resolution because the assumption that climate does not vary at a finer spatial resolution is reasonable, whereas ecological predictors are varying at finer resolutions, and we aimed to characterize the regional differences in elevation. Resampled predictors were informative to the model and contained information that was not captured by other covariates (Figure S1). Other studies have also resolved the mismatch in resolutions of environmental data by resampling predictors to finer (e.g., Duque‐Lazo et al. 2016; Santamarina et al. 2023) or coarser resolutions (e.g., Petitpierre et al. 2017; Pascual‐Rico et al. 2020). We acknowledge resampling predictors to finer resolutions does not increase the accuracy of those predictors but does allow the model to have a more complete suite of covariates.

**FIGURE 1:**
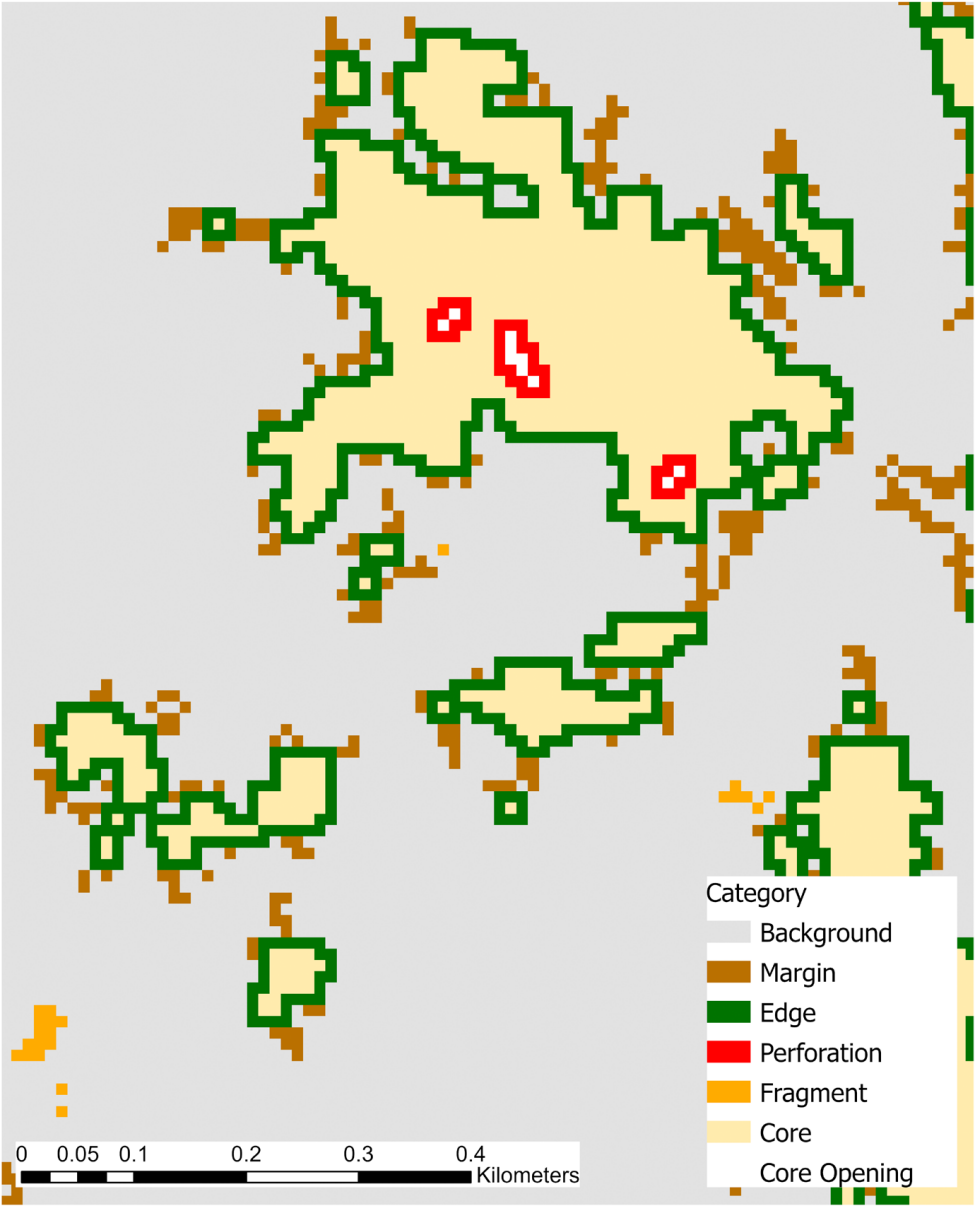
Example of the 7 morphological spatial pattern analysis categories for the combined young forest, shrubland, and understory habitats. Background indicates the absence of young forest, shrubland, and understory vegetation classes. In the foreground, core is interior continuous vegetation classes, edge is the external perimeter of core, fragments are areas too small to contain the core area, and margin is continuous vegetation classes that do not fit into any other category. Core openings are background classes found within core areas, and perforations are the perimeter of those core openings. See Figure 3 for how these categories influence New England and eastern cottontail habitat suitability.

We also used a composite layer of the young forest and shrubland vegetation map and the understory map to run a morphological spatial pattern analysis (MSPA) using the Guidos Toolbox (Soille and Vogt 2009, 2022; Vogt and Riitters 2017; Vogt et al. 2022). We input the composite raster into Guidos Toolbox and set the presence of young forests, shrublands, and understory as the foreground and the absence of those as the background. We used the SPA6 option to classify the foreground into six categories: core, edge, perforation, fragment, margin, and core opening. In the foreground, core indicated interior area, edge was the external object perimeter of the core, perforation was the internal object perimeter of the core, fragment was disjointed areas too small to contain core, margin was non‐contiguous area that did not fit into the other categories, and core opening was the area within perforations (Vogt and Riitters 2017; Vogt et al. 2022). Core opening was the only background class created not from the presence of the input layer, but instead from the arrangement of the foreground around it (Vogt and Riitters 2017; Vogt et al. 2022; Figure 1). The output of the MSPA was included as a categorical predictor in the habitat suitability models.

We used a combination of multicollinearity (Petitpierre et al. 2017) and a priori predictor selection based on ecological knowledge (Zeng et al. 2016) to determine the final list of predictors. Once all predictors determined to have ecological relevance were retrieved, we used pair‐wise Pearson correlations to remove highly correlated predictors (Kuemmerle et al. 2011), |*r*| > 0.50. When a predictor was correlated with multiple predictors, we retained the predictor(s) with the fewest correlations and the greatest biological relevance. We also used a Maxent model run with all predictors to ensure we did not remove predictors that were influential to habitat suitability and to remove any predictors that reduced model gain or performance. This process led to a final set of 15 predictors, which is the average number of predictors used in Maxent models (Bradie and Leung 2017).

### 2.3 Spatial Overlap Analysis Using Habitat Suitability Modeling

To predict suitable cottontail habitat across Connecticut, we ran habitat suitability models in Maxent 3.4.4 (Phillips et al. 2020) for New England and eastern cottontail separately. Maxent is a machine‐learning technique that estimates the unknown probability distribution of a species across both geographic and environmental space using predictors and presence locations, contrasted with the overall distribution within a user‐defined landscape (Phillips et al. 2006). We used cottontail presence data and 15 environmental predictors (14 continuous and 1 categorical predictor) to predict suitable habitat. We used the state of Connecticut as the background extent for the Maxent models due to the availability of fine‐scale spatial data that differentiated vegetation types and spatial patterns (Rittenhouse et al. 2022; Yang et al. 2023), recognizing that Connecticut is a portion of each of the species' range. Previous studies using habitat suitability and niche overlap analyses have also limited the spatial extent to a portion of a species' range when modeling rare or invasive species or when specific areas are of interest (Warren et al. 2008; Kuemmerle et al. 2010; Lioy et al. 2023).

We used a sensitivity analysis to determine the ideal Maxent settings based on the highest area under the receiver operating characteristic curve (AUC) values and ecological knowledge of the species (Merow et al. 2013). We tested regularization parameters of 0.1, 0.5, 1.0, and 2.0, and determined a regularization parameter of 1.0 best reflected the suitable habitat within the study area, based on knowledge of the species and the high AUC values. We used the default of 10,000 background locations (Phillips et al. 2017). We ran models with simple features (linear, hinge, and quadratic), and combinations of those, as well as the auto features function, which evaluated all features (linear, quadratic, product, hinge, and categorical) based on the number of presence records for each species (Phillips et al. 2017). We determined auto features led to higher model performance and captured more of the complex covariate relationships than the simpler features. We chose the logistic output within Maxent, a logistic transformation of the raw maximum entropy values dependent on a prevalence value or the probability a species was present at sites with average conditions (*τ*; Guillera‐Arroita et al. 2014), to report values in terms of relative habitat suitability (Elith et al. 2011). The default *τ* value in Maxent, 0.50, is only suitable for species with similar prevalence values; otherwise, the model error increases, and changing the *τ* value to be more suitable for the species being studied is recommended (Guillera‐Arroita et al. 2014). Occupancy models at the landscape scale found New England cottontail occupancy probability was lower, and eastern cottontail occupancy probability was higher than 0.50; thus, we ran three sets of models for each species so *τ* would reflect the mean (*τ* = 0.25 for New England cottontail, *τ* = 0.79 for eastern cottontail), lower 95% credible interval (*τ* = 0.16 for New England cottontail, *τ* = 0.69 for eastern cottontail) and upper 95% credible interval (*τ* = 0.36 for New England cottontail, *τ* = 0.88 for eastern cottontail) values found in Bischoff et al. (2023a). Specific values reported in the results were from the mean *τ* value model runs, but the output maps were an average of all *τ* values for each species.

We ran a 10‐fold cross‐validation of the model for each *τ* value and species (6 models total, 3 models for New England cottontail and 3 models for EC) to produce error around model estimates and evaluate model performance. We used cross‐validation over selecting training and test data so all the data could be used for model validation (Phillips et al. 2017) and to incorporate randomness into the testing and training data that matches the randomness of the background data (Elith et al. 2011). We used AUC and Test Skill Statistic (TSS) to evaluate the performance of the Maxent models. AUC was calculated within the Maxent software. To calculate TSS, we used the test sample and background predictions (Phillips et al. 2020) to calculate sensitivity and specificity and the maximum test sensitivity plus specificity (MaxSSS) logistic threshold as the threshold for presence/absence (Liu et al. 2013). We determined well‐performing models as AUC values > 0.80 (Hosmer and Lemeshow 2000) and TSS values > 0.60 (Komac et al. 2016).

We created binary suitable habitat maps to measure the spatial overlap between New England and eastern cottontail. We used the MaxSSS logistic threshold (Liu et al. 2013) in Maxent as the threshold for suitable habitat to create binary maps for each species. We averaged the three model outputs for each species and then calculated the percentage of overlap of potential suitable habitat between the two species.

### 2.4 Niche Overlap Analysis Using PCA

In addition to assessing spatial overlap using habitat suitability modeling, we ran an environmental niche overlap analysis using the “ecospat” package (Di Cola et al. 2017) in R 4.3.1 (R Core Team 2023). The niche overlap analysis used an ordination approach with environmental PCA (PCA‐env) to assess how species' niches overlap in environmental space. This was accomplished by projecting the species occurrence density across the entire range of environmental variability found in the study area (Broennimann et al. 2012). The first metric used to measure niche overlap was Schoener's *D* (Schoener 1968). Schoener's *D* varies between 0 and 1, where 0 is complete separation of niches and 1 is complete overlap. Hellinger's *H* is another overlap metric that is used without biological assumptions (van der Vaart 1998). Hellinger's *H* varies between 0 and 2. Thus, an *I* statistic was created instead to have values for *H* on the same scale as Schoener's *D* (Warren et al. 2008). We used both Schoener's *D* and Warren's *I* to measure the degree of niche overlap between the two species.

The niche overlap analysis tests for both niche equivalency and niche similarity, two different hypotheses for comparing niches (Warren et al. 2008; Broennimann et al. 2012). To test for niche equivalency, or whether the two species' niches are equivalent, all occurrence records for each species are pooled and then randomly permutated into two datasets (Warren et al. 2008; Broennimann et al. 2012). This process is repeated 1000 times to get a histogram of simulated *D* and *I* values (Warren et al. 2008; Broennimann et al. 2012). The simulated values were compared to the observed *D* and *I* value, and if the 95% confidence interval for the simulated values did not include the observed values, then the two niches are equivalent (Warren et al. 2008; Broennimann et al. 2012). Niche similarity tests whether the two niches are more similar than what would be expected by chance (Warren et al. 2008; Broennimann et al. 2012). To test for niche similarity, we randomly shifted both species' niches and then recalculated *D* and *I*. We repeated this 1000 times to gain a distribution of simulated *D* and *I* values and calculated 95% confidence intervals (Warren et al. 2008; Broennimann et al. 2012). The two niches were more similar than expected by chance if the 95% confidence interval did not contain the observed value (Warren et al. 2008; Broennimann et al. 2012). The data used for both tests included thinned occurrence records for both species, the 15 environmental predictors, and randomly generated pseudo‐absences. We used the number of pseudo‐absences as a ratio of 10:1 pseudo‐absences to presence records (Dilts et al. 2023).

## 3 Results

### 3.1 Spatial Overlap and Habitat Suitability

**FIGURE 2:**
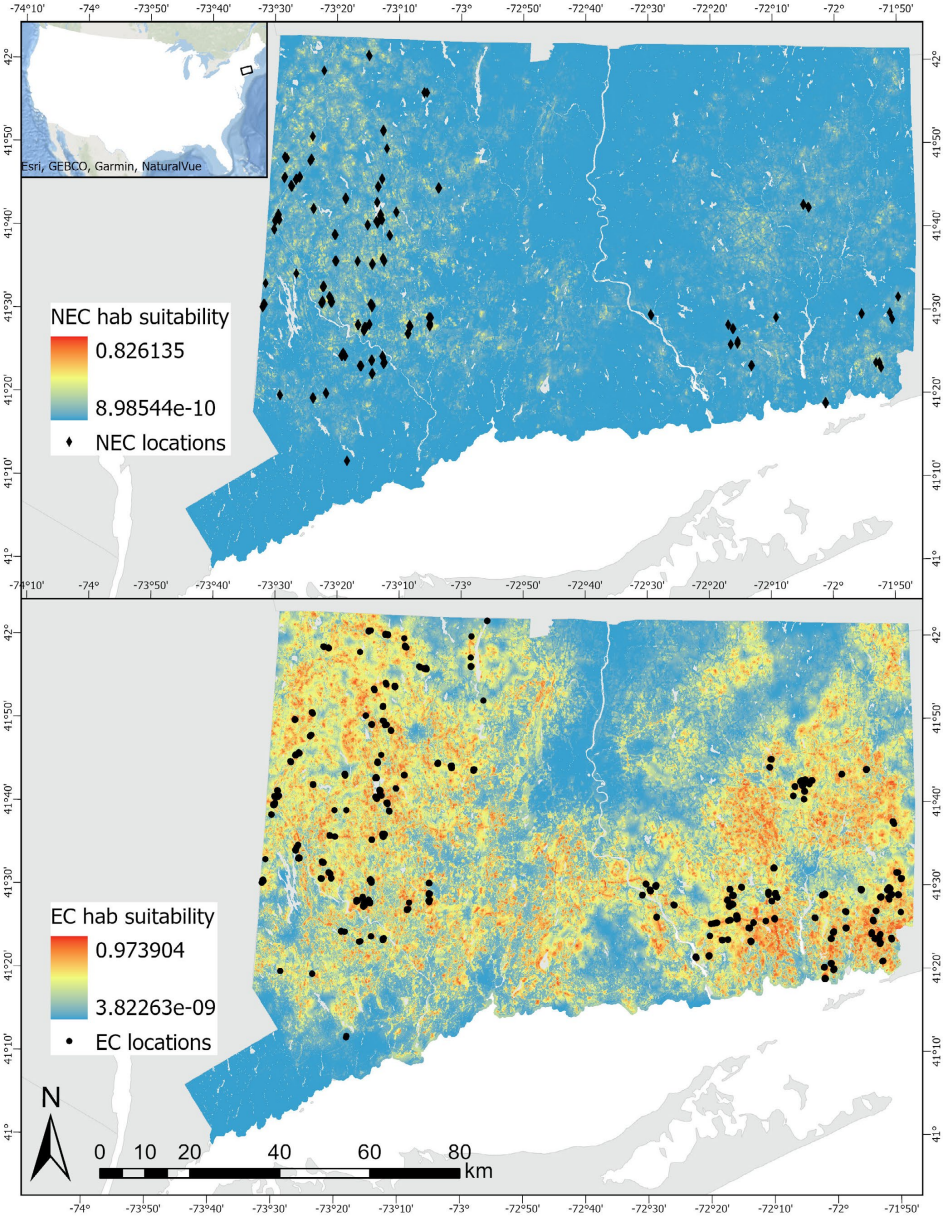
Map of New England cottontail (NEC; black diamonds) and eastern cottontail (EC; black circles) locations in Connecticut used to produce the Maxent outputs. The Maxent outputs shown are an average of the three models, mean, lower, and upper credible interval prevalence values stated in Bischoff et al. (2023a), for each species. The cooler colors indicate lower habitat suitability values, and the warmer colors indicate higher habitat suitability values for the representative species. The projections for the state maps are North American Datum 1983 State Plane Connecticut FIPS 0600 (meters) and the projection for the US map is USA Contiguous Equidistant Conic.

Across Connecticut, the landscape was more suitable for eastern cottontail than for New England cottontail. We found New England cottontail habitat suitability ranged from 0.00 to 0.83 and eastern cottontail habitat suitability ranged from 0.00 to 0.97 (Figure 2). Models for both species performed well, with an average test AUC value of 0.92 (SD = 0.01) and TSS value of 0.89 (SD = 0.02) for New England cottontail models and an average test AUC value of 0.84 (SD = 0.01) and TSS value of 0.77 (SD = 0.01) for eastern cottontail models. In total, we found 83,202.91 ha of potential suitable habitat for New England cottontail, based on the average MaxSSS value of 0.10 for New England cottontail, and 137,496.75 ha of potential suitable habitat for eastern cottontail, based on the average MaxSSS value of 0.64 for eastern cottontail. The percentage of potential suitable New England cottontail habitat overlapping with potential suitable eastern cottontail habitat was 66.11% or 55,004.69 ha.

**FIGURE 3:**
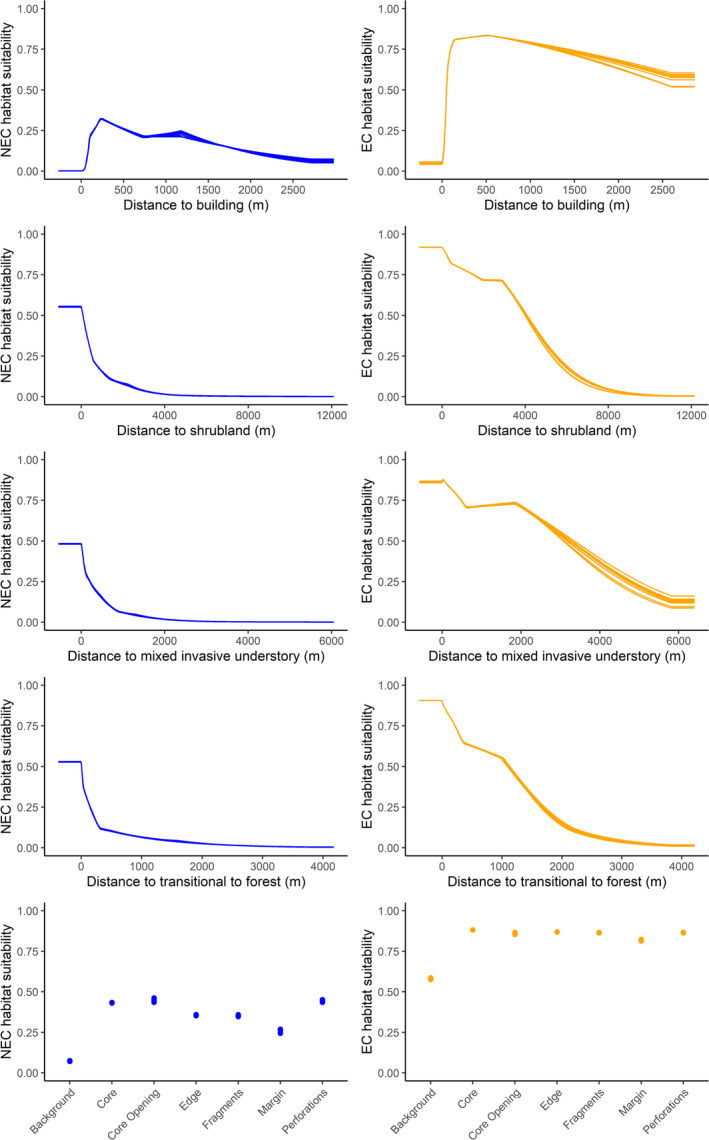
Comparison of New England cottontail (NEC; left column in blue) and eastern cottontail (EC; right column in orange) marginal responses curves to the top 5 contributing predictors in the New England cottontail Maxent model in Connecticut, where the selected predictor was varied while all other predictors were held constant. Each dot or line represents the output of each run in the 10‐fold cross‐validation (10 models for each predictor and species). For the last row of plots, background is area where young forest, shrubland, and understory vegetation classes is absent, core is interior area, edge is the external perimeter of core, fragments are areas too small to contain core area, and margin is continuous area that does not fit into any other category. Core openings are background classes found within core areas and perforations are the perimeter of those core openings.

Both species' habitat suitability was associated with the proximity to and spatial pattern of young forest, shrubland, and understory vegetation. The MSPA predictor had the largest contribution to both the New England (28.34%) and eastern cottontail (27.18%) habitat suitability models (Table 1). The MSPA categories most influential to New England cottontail habitat suitability were core, core openings, and perforations, while all MSPA categories had similar influence on eastern cottontail habitat suitability except background (Figure 3). The land cover most commonly comprising core openings was mixed forest, with 86.63% of the core openings land cover being mixed forest (Table S1). Habitat suitability decreased with increasing distance from shrublands and transitional to forest for both species. However, the contribution of shrublands and transitional to forest differed between the two species, where shrublands contributed more to the New England cottontail model (13.99%) than the eastern cottontail model (2.58%) and transitional to forest contributed more to the eastern cottontail model (25.76%) than the New England cottontail model (9.64%). Understory composition also contributed highly to the habitat suitability for both species, with proximity to a mixed invasive understory contributing more to the New England cottontail model (10.61%) than eastern cottontail (1.28%) while proximity to a native understory species, greenbrier, contributed more to the eastern cottontail model (9.10%) than New England cottontail (6.37%). Distance to buildings also contributed highly to both cottontail species' habitat suitability, 14.38% for New England cottontail and 18.10% for eastern cottontail. New England cottontail habitat suitability was highest at least 250 m away from buildings, while eastern cottontail habitat suitability remained high across all distances to buildings (Figure 3, Table 1).

**TABLE 1:** Percent contribution of the 15 predictors to New England cottontail (NEC) and eastern cottontail (EC) Maxent models in Connecticut.

Predictor	NEC % contribution	EC % contribution
MSPA	28.34	27.18
Building	14.38	18.10
Shrubland	13.99	2.58
Mixed invasive	10.61	1.28
Transitional to forest	9.64	25.76
Greenbrier	6.37	9.10
Barberry	5.55	0.27
Elevation	4.34	1.31
Regenerating forest	3.07	10.05
Mixed forest	1.61	2.78
Precipitation	1.36	0.66
Eastern aspect	0.24	0.06
Forested‐shrub wetland	0.21	0.15
Northern aspect	0.19	0.02
Slope	0.10	0.70

*Note:* The morphological spatial pattern analysis (MSPA) predictor, a categorical predictor, contained background, core, core opening, margin, fragment, edge, and perforation. Background was the area where young forest, shrubland, and understory habitat were absent. In the foreground, core was interior area, edge was the external perimeter of core, fragments were areas too small to contain core area, and margin was continuous area that did not fit into any other category. Core openings were background classes found within core areas and perforations were the perimeter of those core openings.

### 3.2 Niche Overlap

**FIGURE 4:**
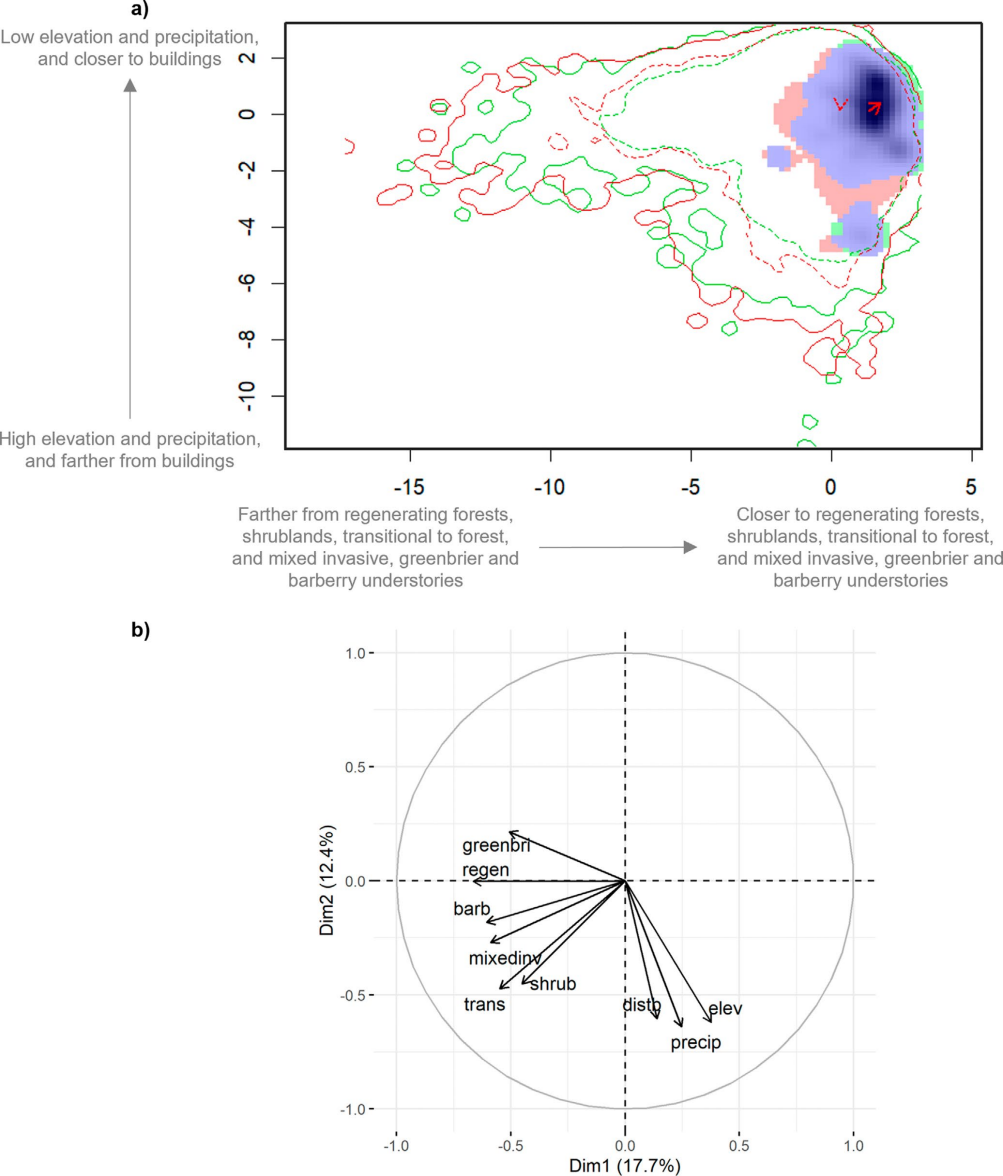
Plots of the niche overlap of New England and eastern cottontail in Connecticut across the two dimensions in the environmental principal component analysis (PCA‐env) including all predictors. In plot a, the blue‐colored area is the area where eastern cottontail niche overlaps with the New England cottontail niche. The red‐shaded area represents where eastern cottontail niche does not include New England cottontail niche, the green‐shaded area represents where New England cottontail niche does not include eastern cottontail niche. The solid lines represent niches within all available environmental conditions, while the dotted lines represent niches within 50% of the available environmental conditions (New England cottontail is green, eastern cottontail is red). The darker shading represents the occurrence density of New England cottontail within its own range. The red arrows represent the shift in niche centroids between New England cottontail and eastern cottontail niche, where the red solid arrow shows the shift in environmental conditions and the dotted red arrow shows the shift in background conditions between the two species' niches. Plot b includes the percentage of each dimension of the PCA‐env explaining the variation in predictors and the top 7 contributing predictors to the PCA‐env: Annual precipitation (precip), regenerating forest (regen), elevation (elev), shrubland (shrub), transitional to forest (trans), and barberry (barb) and mixed invasive understory (mix_inv). The interpretations of the dimensions in plot a correspond to the predictors shown in plot b.

**FIGURE 5:**
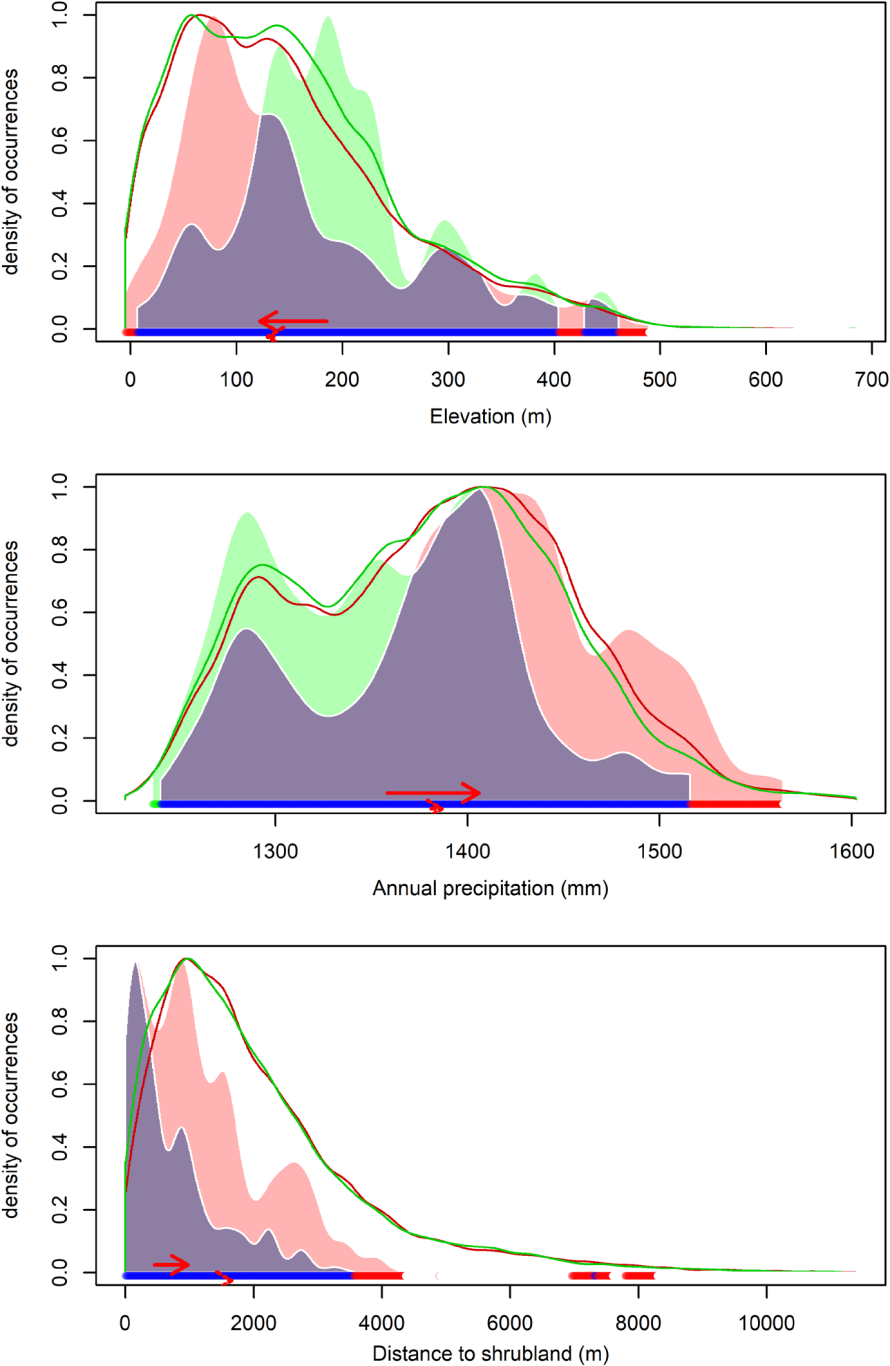
Density plots of niche overlap for New England cottontail and eastern cottontail across the range of the 3 environmental predictors with niche shifts: elevation, annual total precipitation (mm), and distance to shrublands. For all plots, the gray‐shaded area and blue bar represent the overlapping of the two species' niches, the red‐shaded area and bar represent the eastern cottontail range, and the green‐shaded areas and bar represent the New England cottontail range. The solid lines represent the extent of the species niche across the range of the predictor values. The red solid arrow shows the niche centroid shift (in favor of eastern cottontail) in environmental conditions and the dotted red arrow shows the shift in background conditions between the two species' niches.

New England cottontail and eastern cottontail environmental niches had high overlap (*D* = 0.79, *I* = 0.89). We found support that niches had more overlap than random, with *D* both more similar (*p*‐value < 0.01) and equivalent (*p*‐value = 0.01) than random and *I* more similar than random (*p*‐value = 0.01). We did not find support that the niches were more equivalent than random for the *I* value (*p*‐value = 1). We found niche overlap for New England cottontail and eastern cottontail changed minimally with current environmental conditions (Figure 4a). The PCA‐env explained 30.10% of the variation observed in the predictors with dimension 1 accounting for 17.67% and dimension 2 accounting for 12.43% of the variation (Figure 4b). The first dimension largely represented a gradient of increasing proximity to young forest and understory vegetation represented by negative correlation values with distance to regenerating forest, transitional to forest, and barberry, mixed invasive, and greenbrier understories (*r* = −0.66, −0.55, −0.61, −0.59, −0.51). Dimension 2 largely represents a gradient of decreasing annual precipitation, elevation, and distance to buildings represented by negative correlation values (*r* = −0.64, −0.62, −0.60; Table S2). We found that varying annual precipitation, elevation, and distance to shrublands did show niche shifts, where annual precipitation below 1375 mm, elevation above 125 m, and adjacent to shrublands favored New England cottontail (Figure 5), while all other predictors did not demonstrate niche shifts (Figure S2).

## 4 Discussion

**FIGURE 6:**
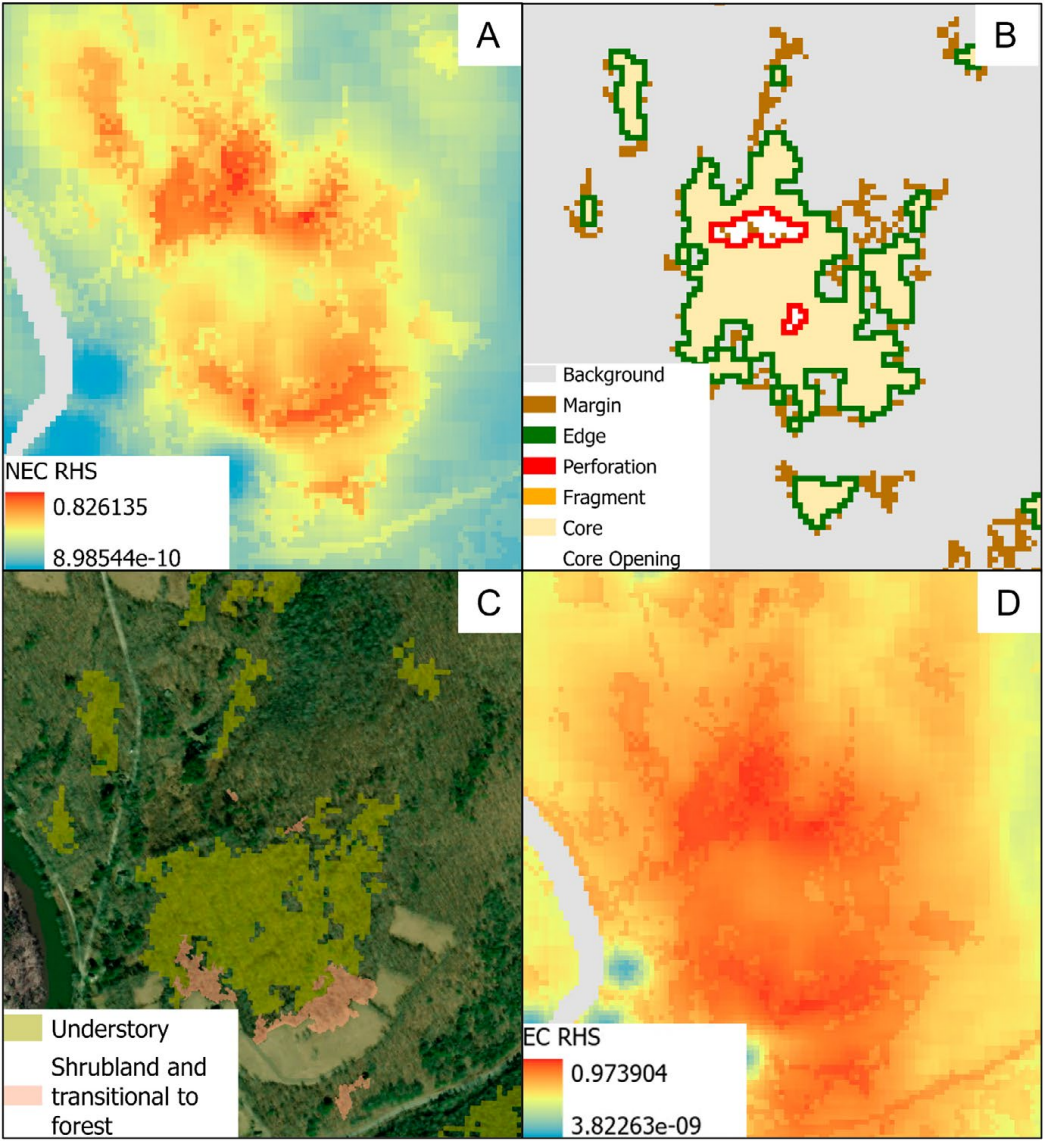
Comparison of New England cottontail (NEC) Maxent output (A), morphological spatial pattern analysis output (B), presence of understory species, shrublands, and transitional to forest (C), and eastern cottontail (EC) Maxent output (D) in a specific area of highly suitable New England cottontail habitat in Connecticut. For the Maxent outputs, the warmer colors indicate a higher relative habitat suitability value (RHS) for the species, and the cooler colors indicate a lower RHS value for the species. For the morphological spatial pattern analysis, background is the absence of young forest and shrubland vegetation, core is continuous interior young forest and shrubland vegetation, edge is the perimeter of core, fragment is a continuous area too small to be core, core opening is background classes found within core, perforation is the perimeter of core openings, and margins are young forest and shrubland vegetation that does not fit into any other classification. Understory indicates the presence of both native and invasive deciduous understory species. Shrublands is a young forest class created through the process of succession (no recent forest disturbance) with vegetation height mostly between 0.5 to 2.5 m, and transitional to forest is also created through the process of succession but within a mixture of vegetation height above and below 2.5 m.

Understanding the overlap in the suitable habitat (i.e., spatially) or niches (i.e., environmentally) between native and introduced species can help determine the potential spread of invasive species and conserve habitat for rare species (Santamarina et al. 2023). Overall, we found high overlap between the suitable habitat and niches of New England and eastern cottontails, where the highly suitable New England cottontail habitat was also highly suitable for the eastern cottontail, and the two cottontail niches were statistically more similar than random yet not equivalent. We observed some covariates that could reduce the overlap in niches and suitable habitat. Sites of higher elevation and lower precipitation shifted niches in favor of the New England cottontail and led to less overlap with the eastern cottontail niche. Proximity to shrublands and mixed invasive understory was more important for New England cottontail habitat suitability than for eastern cottontail habitat suitability. A novel finding was the importance of the spatial arrangement of vegetation types for New England cottontail habitat suitability, specifically patches of mature forest without understory intermixed within complexes of young forest, shrubland, and understory habitat. Thus, vegetation management that focuses on habitat heterogeneity may create new patches or increase the suitability of existing patches for the New England cottontail (Figure 6). However, these habitat conditions did not alter the niche overlap or negatively influence eastern cottontail habitat suitability; thus, vegetation management alone may not be enough to discourage eastern cottontail populations. Direct species management options, such as strategic eastern cottontail removal, should be explored, especially as future conditions, such as higher predicted precipitation totals (Jong et al. 2023) and increased urbanization, will likely further favor the eastern cottontail niche.

We report high winter‐suitable habitat and niche overlap for the native New England cottontail and its congener, the introduced and range‐expanding eastern cottontail. We focused on winter presence records because of the high‐quality data produced by the New England Cottontail Regional Monitoring Program, with its standardized and consistent protocol. The regional monitoring data had high detection (Brubaker et al. 2014; Bischoff et al. 2023a) because pellets were more visible on snow and cold conditions retained sample quality (Kovach et al. 2003; Brubaker et al. 2014; Whipps et al. 2020). During winter, New England cottontail survival is low and competition is high (Cheeseman et al. 2018, 2021); thus, our results reflect suitable habitat during the time of year when the ecological burden is highest. Although including summer cottontail occurrence data may yield differences in habitat suitability because habitat selection, survival, and competition for cottontails can vary seasonally (Cheeseman et al. 2018, 2021; Cheeseman, Cohen, Ryan, et al. 2019; Kilpatrick and Goodie 2020), our results shed new light on the importance of intermixing young forest, shrubland, and understory habitats within mature forest for New England cottontail.

### 4.1 Habitat Suitability

From our study, we found that the importance of young forest and shrublands for cottontail habitat suitability largely reflects what is known about the habitat associations of the two species in the northeastern United States. Both species' suitable habitat was influenced by the presence of young forests, shrublands, and understory (Cheeseman et al. 2018, 2021; Cheeseman, Cohen, Ryan, et al. 2019; Bischoff et al. 2023a; Bischoff et al. 2023b). Specifically, proximity to shrublands increased New England cottontail habitat suitability, which increased New England cottontail occupancy and colonization at the landscape scale (Bischoff et al. 2023a) and abundance at the patch level (Bischoff et al. 2023b). However, we found not only is the proximity to these vegetation types influential to New England cottontail habitat suitability, but the spatial arrangement of these vegetation types contributed most to habitat suitability for New England cottontail. Specifically, patches of mature forest without understory components within complexes of young forest, shrubland, and mature forest with understory components increased New England cottontail habitat suitability the most. This agrees with many studies that have demonstrated the importance of high vegetation height and canopy closure for New England cottontail, especially in patches co‐occupied by eastern cottontail (Buffum et al. 2015; O'Connor 2015; Cheeseman et al. 2018, 2021; Cheeseman, Cohen, Ryan, et al. 2019; Bischoff et al. 2023a; Bischoff et al. 2023b) and a recent study that found New England cottontail increased the proportion of their home range that included vegetation management areas if some canopy was retained (Eline, Cohen, Whipps, et al. 2023). Because of these findings, guidance for New England cottontail habitat management suggests utilizing shelterwood cuts and leaving residual trees to maintain vegetation height and some canopy closure (Cheeseman and Cohen 2019). Our study supports these recommendations but adds that residual trees clumped in small patches within young forest and shrubland habitat may enhance existing patches and create new patches suitable for New England cottontail. These recommendations and results are applicable for New England cottontail in Connecticut, a heavily forested landscape where heterogeneity may be created through removal of mature forest trees but may not be applicable where New England cottontail occupies less forested habitats, such as the coastal areas in Maine and Massachusetts.

Despite the benefits of habitat management to New England cottontail, focusing on vegetation management alone will likely not be enough to discourage eastern cottontail populations. We found that the overlap of suitable habitat between New England and eastern cottontail was high regardless of the spatial arrangement of habitat and the distance to young forest, shrubland, and understory habitat. Higher vegetation height was found to decrease eastern cottontail survival and occupancy in several other studies (Buffum et al. 2015; O'Connor 2015; Cheeseman et al. 2018, 2021; Cheeseman, Cohen, Ryan, et al. 2019; Bischoff et al. 2023a; Bischoff et al. 2023b). Vegetation management within strategic areas is crucial for New England cottontail management to establish connectivity between populations (Ferry 2023) but may need to be coupled with population augmentation or translocation to sustain New England cottontail populations (Bauer et al. 2020). Other states have found coupling habitat connectivity and population augmentation to be a successful strategy for maintaining New England cottontail populations (Bauer et al. 2020). Another potential management action is the removal of eastern cottontail to limit or reduce the expansion of the species in suitable New England cottontail habitat. The removal of eastern cottontail was tested at a few patches in Connecticut but was not effective in reducing eastern cottontail abundance (Kilpatrick n.d., unpublished data). The removal of invasive mammalian species has long been a management practice for problematic species and has largely been successful, but the success depends on the predator response to species removal (Norbury 2001), consistent effort of the program, and controlling large areas (Robertson et al. 2017). The removal of eastern cottontail would contain many complexities and considerations. Eastern cottontail has been present in the northeastern United States for over 100 years (Johnston 1972) and has successfully expanded across most of the region. Additionally, the mechanism for removal may be difficult because cottontail hunting is not common in the state and cottontail trapping success is relatively low (Cheeseman et al. 2021). Furthermore, New England and eastern cottontail can only be differentiated confidently through genetic analysis, which requires large amounts of time and resources. Despite these challenges, strategic removal of eastern cottontail in newly managed areas for New England cottontail may offer New England cottontail the advantage of colonizing habitat before eastern cottontail. Control of eastern cottontail may be especially relevant in the northern extents of the New England cottontail range, where eastern cottontail prevalence is lower and the time since eastern cottontail invasion is several decades less than the southern extent of the New England cottontail range.

Predicting New England cottontail suitable habitat across the state is crucial for planning New England cottontail conservation, management, and monitoring for the future. Some of these highly suitable areas for New England cottontail have not been sampled in recent years; thus, areas of highly suitable New England cottontail habitat with limited occurrence data provide opportunities for identifying new populations, locations for eastern cottontail removal, and potential sites for habitat enhancement or New England cottontail reintroductions. The maps of suitable habitat also allow further direction for managing New England cottontail in the two regions of Connecticut where New England cottontail is extant: the eastern and western portions of the state. Differences in New England and eastern cottontail populations in eastern and western Connecticut have been found in other studies (Kristensen and Kovach 2018; Bischoff et al. 2023b). Specifically, the low habitat suitability for New England cottontail and generally high habitat suitability for eastern cottontail in eastern Connecticut is evidence that explains the decline in New England cottontail relative abundance observed in the eastern portion of the state over the past few years (Bischoff et al. 2023b). Identifying new areas suitable for New England cottontail in the region of the state where New England cottontail occupancy is not declining will provide enhanced opportunities for management and possibly provide more connections between isolated patches currently occupied by New England cottontail. We argue that additional conservation actions, including augmentation and translocation of New England cottontail, vegetation management to establish, enhance, or maintain inter‐mixed habitats, and consideration of eastern cottontail removal may be needed to benefit New England cottontail.

### 4.2 Niche Overlap Analysis

New England and eastern cottontail had unusually high niche overlap and low niche differentiation for two species. Niche overlap analyses with other species have found a mix of both high and low niche overlap (Liu et al. 2017; Pascual‐Rico et al. 2020; Quiroga and Souto 2022), but few studies with two mammalian species have found similarly high niche overlap values and low proportions of the native species niche not overlapping with the introduced species niche. Although New England and eastern cottontail niches were highly similar, they were not equivalent. The lack of equivalency suggests outside conditions or pressures may influence the prevalence of eastern cottontail across the state despite high niche overlap with New England cottontail.

One possible mechanism for the high observed overlap in environmental space, but not geographic space, is competition. Previous studies have identified competition between the two species and supported eastern cottontail as the dominant competitor (Probert and Litvaitis 1996; Cheeseman et al. 2018; Bischoff et al. 2023b). Competition between New England and eastern cottontail was first observed in behavioral studies but lacked evidence of interference competition (Probert and Litvaitis 1996). A resource selection study of radio‐tracked individuals associated interspecific competition with the displacement of New England cottontail from young forest and shrubland habitat (Cheeseman et al. 2018). Relative abundance studies further supported interspecific competition, where patches with high numbers of eastern cottontail had lower New England cottontail abundance (Bischoff et al. 2023b). A rigorous experimental test of New England cottontail–eastern cottontail competition, during all seasons and parts of the annual life cycle, would confirm the mechanism(s) and provide insight into paths for New England cottontail conservation in the continued face of eastern cottontail range expansion.

## Author Contributions

**Kathryn E. Bischoff:** conceptualization (equal), data curation (equal), formal analysis (equal), methodology (equal), software (equal), validation (equal), visualization (equal), writing – original draft (equal), writing – review and editing (equal). **Danielle Katz:** conceptualization (equal), data curation (equal), formal analysis (equal), methodology (equal), software (equal), validation (equal), visualization (equal), writing – original draft (equal), writing – review and editing (equal). **Chadwick D. Rittenhouse:** conceptualization (equal), funding acquisition (equal), methodology (equal), supervision (equal), visualization (equal), writing – original draft (equal), writing – review and editing (equal). **Tracy A. G. Rittenhouse:** funding acquisition (equal), supervision (equal), writing – review and editing (equal).

## Conflicts of Interest

The authors declare no conflicts of interest.

## Supporting information


Data S1

## Data Availability

The data used in the analysis of this study are available through Dryad at https://datadryad.org/stash/share/BMmVlIBYahrOw‐_pzsw‐_hPC0w2oyhG3F2EntvXG2jg. To prevent poaching and harm to New England cottontail, the species location data provided are at a coarser resolution (1.0 km), but covariates are measured at the resolution used within the analysis.
