# Supplementary material for: High Overlap in Niches and Suitable Habitat Between an Imperiled and Introduced Cottontail
Source: Ecol Evol. 2025 Mar 17;15(3):e71083. doi: 10.1002/ece3.71083 (PMC11917141; doi:10.1002/ece3.71083)
Supplement: Supplementary file 1 — Data S1 [file ECE3-15-e71083-s001.docx]

Supporting Information

High overlap in niches and suitable habitat between an imperiled and introduced cottontail

Table S1. Proportions of land cover types comprising all the core openings within Connecticut. Core openings were determined using morphological spatial pattern analysis and defined as background classes found within continuous interior vegetation classes.

| Landcover class | Proportion |
| --- | --- |
| Background | <0.01 |
| Impervious development | 0.01 |
| Developed open space | 0.01 |
| Cultivated land | <0.01 |
| Pasture/hay | <0.01 |
| Grassland | 0.02 |
| Mixed forest | 0.87 |
| Scrub/shrub | 0.01 |
| Palustrine forested wetland | 0.06 |
| Palustrine scrub/shrub wetland | 0.01 |
| Palustrine emergent wetland | 0.01 |
| Bare land | <0.01 |
| Open water | 0.01 |
| Palustrine aquatic bed | <0.01 |

Table S2. Summary of correlations for each predictor for dimension 1 and 2 in the PCA. The morphological spatial pattern analysis (MSPA) predictor contained background, core, core opening, margin, fragment, edge, and perforation.

| Predictor | Dimension 1 | Dimension 2 |
| --- | --- | --- |
| Building | 0.14 | -0.60 |
| MSPA | 0.31 | -0.16 |
| Barberry | -0.61 | -0.18 |
| Elevation | 0.38 | -0.62 |
| Forested-shrub wetland | -0.41 | -0.05 |
| Greenbrier | -0.51 | 0.22 |
| Mixed invasive | -0.59 | -0.27 |
| Mixed forest | -0.43 | 0.13 |
| North | -0.10 | 0.01 |
| East | -0.01 | -0.04 |
| Regenerating forest | -0.66 | 0.00 |
| Shrubland | -0.45 | -0.45 |
| Slope | 0.24 | -0.28 |
| Transitional to Forest | -0.55 | -0.47 |
| Precipitation | 0.25 | -0.64 |

Figure S1. Jackknife plots demonstrating the effect of each predictor on test and training gain, and AUC for New England cottontail (NEC) and eastern cottontail (EC) Maxent models.


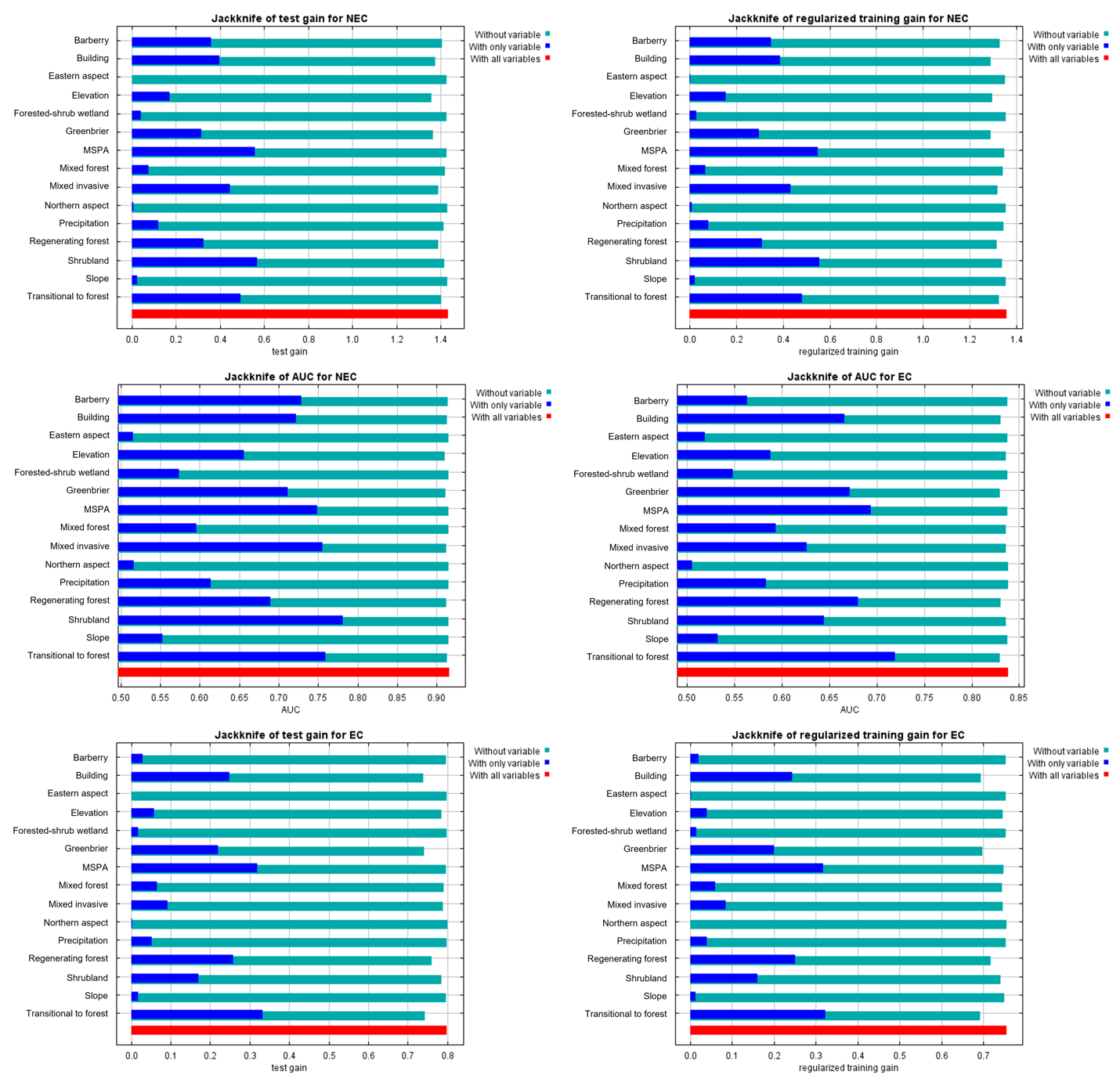


Figure S2. Density plots of niche overlap and niche dynamics across the range of each environmental predictor. The grey shaded area and blue bar represent the overlapping of New England and eastern cottontail niches, the red shaded area and bar represents the eastern cottontail range, and the green shaded areas and bar represent the New England cottontail range. The solid lines represent the extent of the species niche across each predictor value range. The red solid arrow represents the niche centroid shift (in favor of eastern cottontail) in environmental conditions and the dotted red arrow shows the shift in background conditions between the two
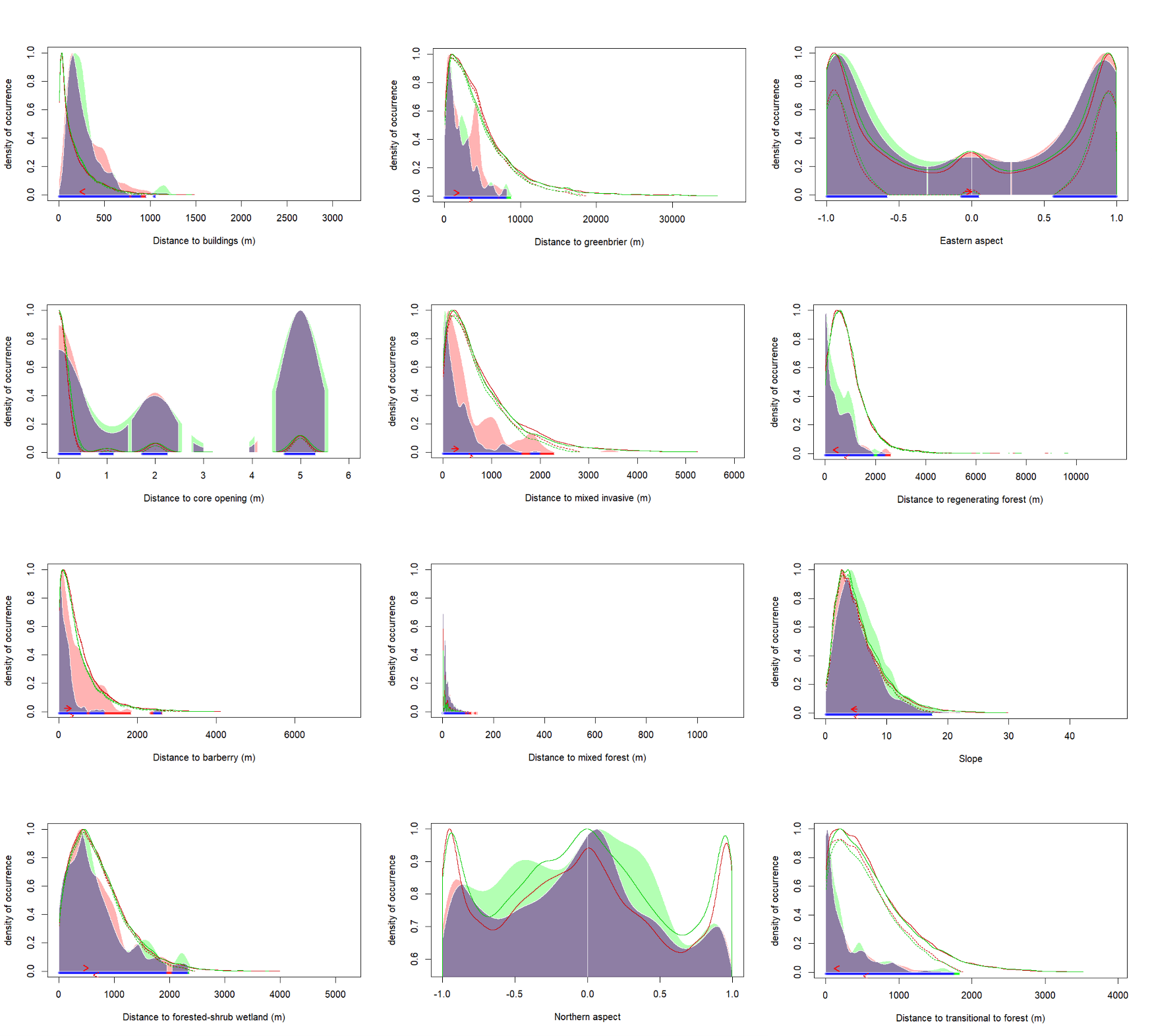
species’ niches.
